# Case report of undiagnosed retrosternal goitre – an unpleasant finding during complex cardiac surgery procedure

**DOI:** 10.1186/1749-8090-8-S1-P31

**Published:** 2013-09-11

**Authors:** Igor Medved, Marin Oštrić, Salem Osman, Aleksandra Ljubačev, Alfred Božić

**Affiliations:** 1 Department of Cardiac Surgery, Clinical Hospital Center Rijeka, Rijeka, Croatia; 2 Department of Anesthesiology and Intensive Care, Clinical Hospital Center Rijeka, Rijeka, Croatia

## 

An 84-years old patient was scheduled for elective, combined procedure of aortic valve replacement and coronary artery bypass grafting. After median sternotomy was done, there was a finding of unusual retrosternal mass. Concurrently, otherwise routine procedure, changed it course. During surgical manipulation, patient started to show signs of thyreotoxic crisis.

Patient was initially stabilised with intravenous metoprolol and amiodarone, blood was taken for analysis and we decided to continue with planned procedure.

Bioprosthesis and vein graft on PD were implanted. During operation , blood analysis came through and revealed high levels of FT4 – free thyroxin : 44,10 pmol/L (with normal values of 11,5 – 22,7 pmol/L).

After CPB termination and adequate hemostasis, with help of general surgeon, partial thyreoidectomy was done.

In early postoperative course, patient recived some inotropic support and was extubated on 4th postoperative day. Except for some minor restlessness, recovery went uneventfull and 84 years old patient was released in good health, on 18th postoperative day.

Upon release, patient was reffered to endocrinologist for further treatment.

